# Historical milestones in renal pathology

**DOI:** 10.1007/s00428-012-1254-7

**Published:** 2012-06-03

**Authors:** Jan J. Weening, J. Charles Jennette

**Affiliations:** 1 Department of Pathology, Erasmus MC University Medical Center, Rotterdam, The Netherlands; 2 Department of Pathology, Tergooiziekenhuizen, Hilversum, The Netherlands; 3 Department of Pathology and Laboratory Medicine, University of North Carolina at Chapel Hill, Chapel Hill, NC USA

## Introduction

The history of renal pathology can be divided into two eras: one starting with the invention of the microscope and its application to renal tissue; the second with the introduction of the renal biopsy, which coincided with the development of electron microscopy and immunofluorescence microscopy that allowed the analysis of pathological changes in great detail with an eye for pathogenesis and pathophysiology. These two eras started in 1650 and 1950, respectively; an interval of no less than three centuries during which clinicians and pathologists struggled with a lack of clear understanding of the causes and evolution of renal disease. In concordance with the design of this series on the history of pathology, we focus primarily on the history of renal pathology before 1970.

**Table 1:** Time table of seminal discoveries in renal pathology

1666	The glomerulus	[1]
1842	The nephron	[3]
1827	Clinical renal syndromes	[4]
1914	First renal histopathology classification	[15]
1900–1950	Experimental autoimmune glomerulonephritis	[25, 26]
1950–1960	Renal biopsy	[29–32]
1950–1970	Immunofluorescence microscopy, electron microscopy	[33–37]
1960–present	Immune complex diseases, anti-GBM, lupus nephritis, post-infectious GN, IgAN	[27, 28, 42–47, 51]
1975–present	Focal segmental glomerulosclerosis	[19–21, 58, 59]
1980–present	ANCA disease	[54–56]
1980–present	Membranous glomerulopathy pathogenesis	[48–50]
1990–2009	Hemolytic uremic syndrome	[60]
1990–present	Podocyte pathobiology	[57, 59]
1990–present	Classification of diseases of the transplanted kidney	[62]

## The early phase

### Renal histology

**Fig. 1:**
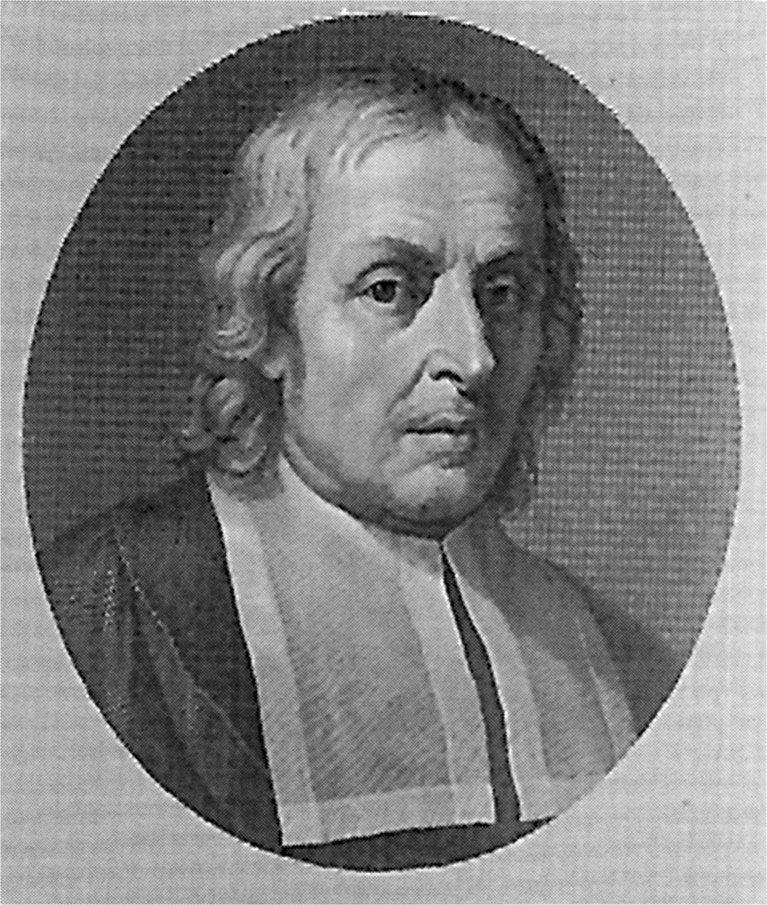
Marcello Malpighi, approx 1660. From the Istituto e Museo di Storia della Scienza, Museo Galileo, Firenze, Italy

The origins of renal pathology can be traced back to the earliest descriptions of the microanatomy of the kidney by Marcello Malpighi in the year 1666 [1]. Based on his observations with the newly developed microscope, Malpighi (1628–1694; Fig. 1) hypothesized that the formation of urine took place in the kidneys through a filtering mechanism between blood and renal tubules [1]. Malpighi had studied medicine in Bologna and was professor in medicine first in Pisa and then at his alma mater and at the academy of Messina where he published his microscopic observations of various organs including the kidney. Before his illuminating studies, the relation between the kidney and the excretion of urine had been unclear and a subject of wild speculation as was true for renal function and disease in general.

The microscope was a new device at that time used by only a handful of researchers including Malpighi, Antoni van Leeuwenhoek, and Robert Hooke. Hooke had made an improved version of the instrument that was used by his Italian colleague. In combination with a talent for drawing and a sharp inventive mind, Malpighi was able to make giant strides forward, notwithstanding opposition from many colleagues at the time.

**Fig. 2:**
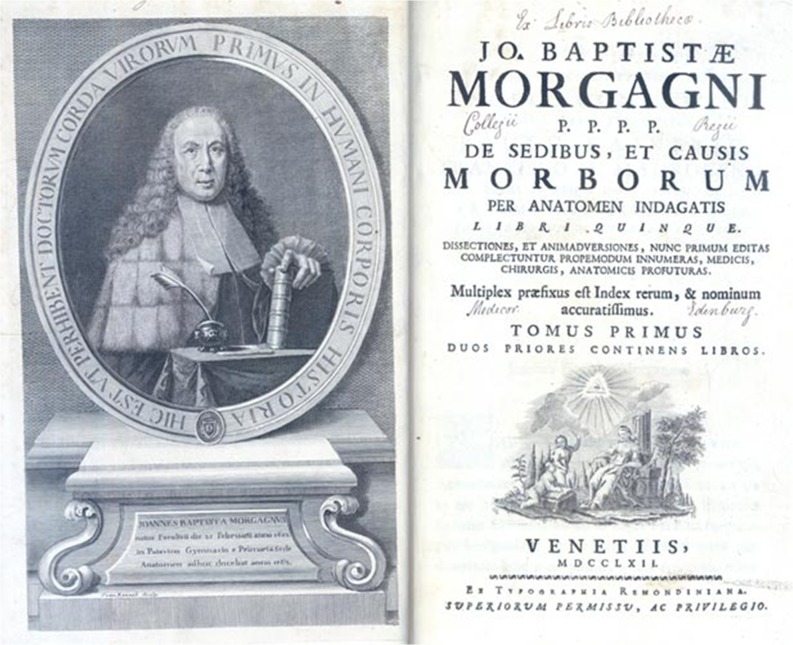
Giovanni Baptista Morgagni’s major work “DE SEDIBUS ET CAUSIS MORBORUM” (Ed. Venice, 1762). From the Bibliotheca Accademia Pugliese, Italy

In spite of the seminal contributions of Malpighi based on the use of the microscope, his findings were completely overlooked by Giovanni Battista Morgagni who is regarded as the father of pathological anatomy. Morgagni was born in 1682 and lived and worked immediately after Malpighi, who had died in 1694. Morgagni was an anatomist and clinician and related postmortem findings to his clinical observations. He performed over 600 autopsies that form the basis for his major work *De Sedibus et causis morborum per anatomem indagatis* that was published in 1761 (Fig. 2) and had many reprints throughout Europe [2]. However, a complete understanding of the various diseases described in this book including those of the kidneys failed because of a lack of microscopic analysis, which Morgagni refused to rely on as an important tool.

**Fig. 3:**
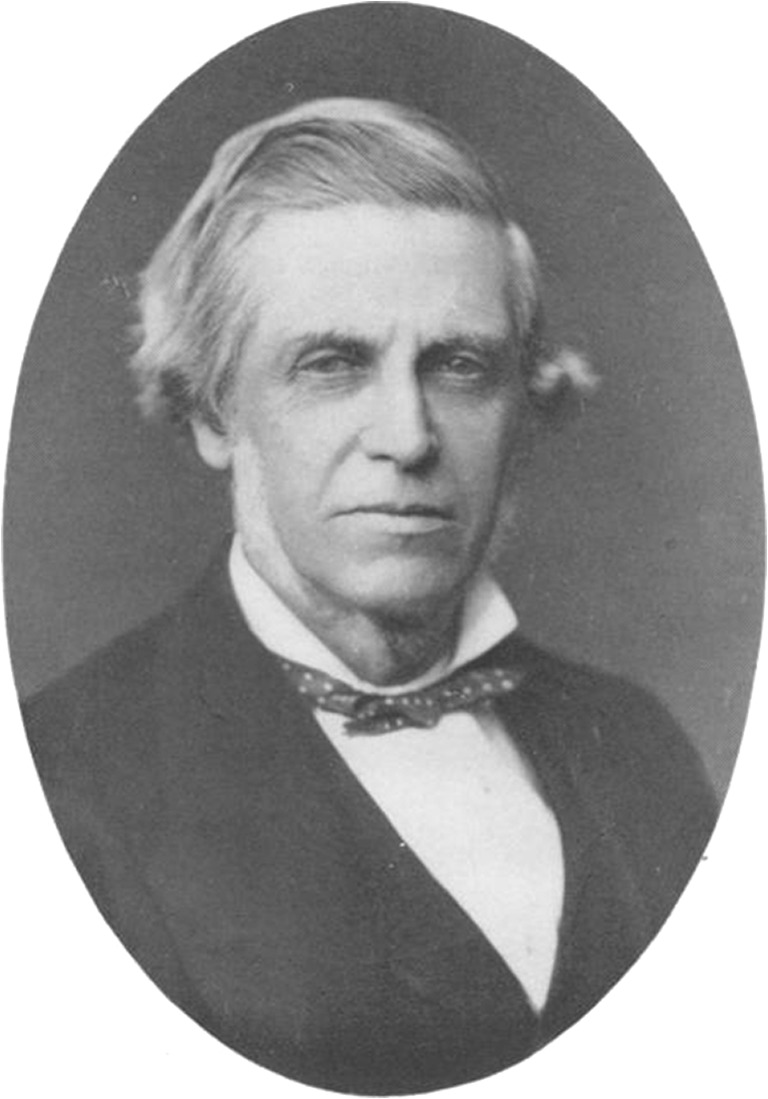
Sir William Bowman, approx 1875, From the Science Photolibrary

**Fig. 4:**
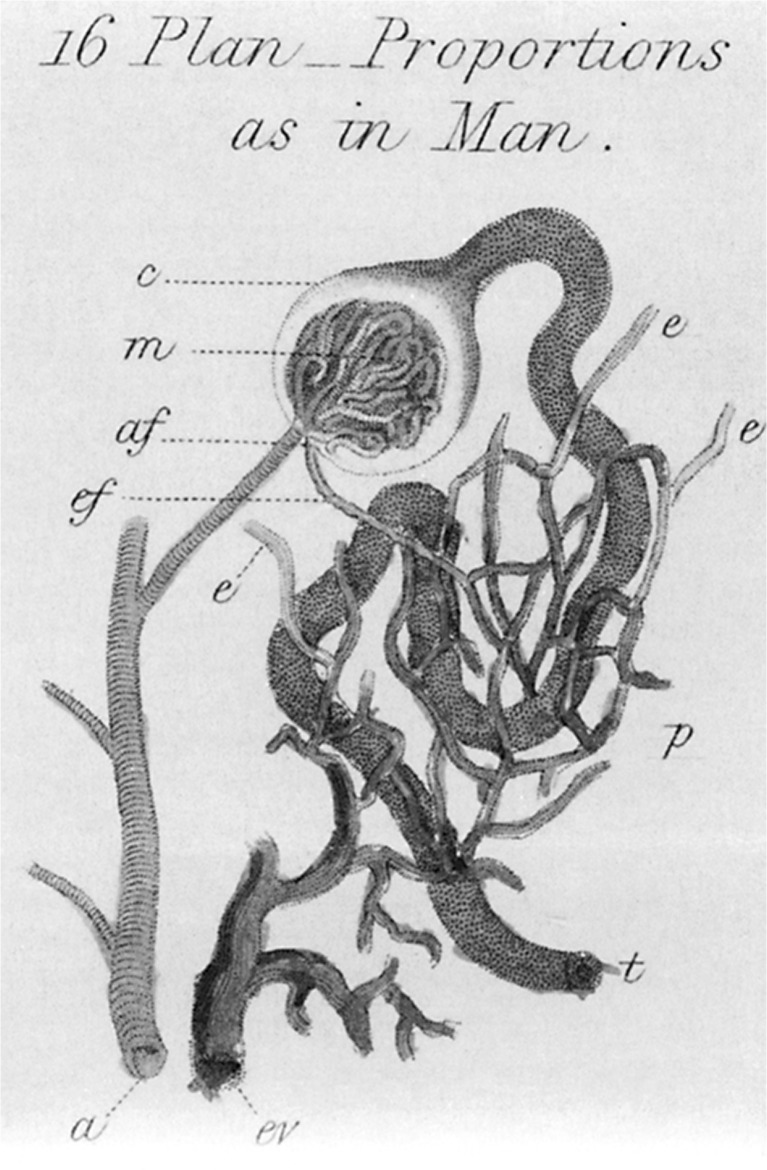
Bowman’s schematic drawing of the nephron. From The Science Photolibrary

Malpighi’s work was taken to a higher level by William Bowman, an English physician (1816–1892) who would later become a renowned ophthalmologist but devoted his early scientific life to the study of anatomy and physiology of the human body. One of his major contributions was the description of the components of the proximal part of the nephron with the periglomerular capsule (later named after him) as it is connected to the proximal tubule allowing the escape of filtrate produced by the glomerular capillary network (Figs. 3 and 4) [3].

### Clinical nephrology

During the time of Bowman’s early work, Richard Bright (1789–1858) together with John Bostock in England, Pierre Francois Rayer in France, and Friedrich Theodor von Frerichs in Germany studied patients with kidney diseases in great detail by correlating clinical symptoms and chemical derangements in blood and urine [4–7]. Pathology was limited to macroscopy and its contribution to diagnosis was therefore limited. Edema (dropsy as it was termed by Bright), hypertension, hematuria, oliguria, albuminuria, and uremia were quite well described but the underlying processes remained obscure. We now know that these symptoms can be caused by primary or secondary renal disease, affecting the glomeruli, the tubules, or the interstitium based on many different underlying events, which can only be recognized by microscopic analysis. The philosophy held at that time, however, was that in disease, functional alterations led to visible changes, and the latter, being secondary, were considered of less importance.

Richard Bright described three main macroscopic appearances of the diseased kidneys and tried to classify renal diseases accordingly [4] but admitted that he did not know whether these were mere stages of one disease or the faces of many different diseases from acute inflammatory to degenerative to sclerotic. Carl von Rokitansky, the famous pathologist from Vienna (1809–1878), took this to an extreme and used no less than eight classes of renal disease in albuminuric patients, one of which was renal amyloidosis [8]. Clearly, until microscopic analysis was applied to the pathological diagnosis of patients with various clinical syndromes, a rational classification of renal diseases was impossible.

### Renal pathology (1850–1900)

The contribution of pathology was on its way, but it took many steps to achieve a level of refinement necessary to understand what is really going on in diseased renal tissues allowing a precise diagnosis of renal disease. Microscopic evaluation of renal tissue was facilitated by a number of technical advances in the mid nineteenth century. In 1837, Gabriel Valentin, a Swiss–German professor of physiology at Bern University, developed the technique of making thin tissue slides. Valentin described renal histology in patients who had died from massive proteinuria [9]. He observed no changes in the corpora Malpighia, but extensive accumulation of a grayish substance in the tubules soon to be recognized as fat, which subsequently led to the term lipoid nephrosis. In 1854 as a result of the invention of aniline dyes, more stains became available in addition to carmine, the only tissue stain that could be used until that year. Edwin Klebs, a pathologist who had worked with Virchow in Berlin, but had moved on to Switzerland and later to the US, introduced paraffin embedding in 1869 and used his improved histologic preparations to study renal disease. He coined the term glomerulonephritis in his pathology textbook [10].

The field then expanded quickly and from different sources evidence accumulated that pathological changes varied considerably between patients with similar “classes” of kidney disease. Based on microscopic analysis, several renal diseases were specifically identified including post-scarlatina glomerulonephritis, renal amyloidosis, and nephrosclerosis.

### Cellular pathology

Important for the unraveling of tissue injury was the concept of cellular injury. The view of the cell as the basic unit of structure and function in the human body was first reported by Robert Remak [11], a Polish Jewish scientist, based on his observations that cell division led to cell renewal. In 1858 after first opposing this view, Rudolph Virchow applied it to his concept of cellular pathology [12], although he did not acknowledge the seminal work of Remak. The concept of cellular pathology was as valid for the kidney as for any other organ, but it took over a hundred years to unravel the characteristics of the variously differentiated cells of the nephron and their role in renal pathology.

### Cellular physiology

**Fig. 5:**
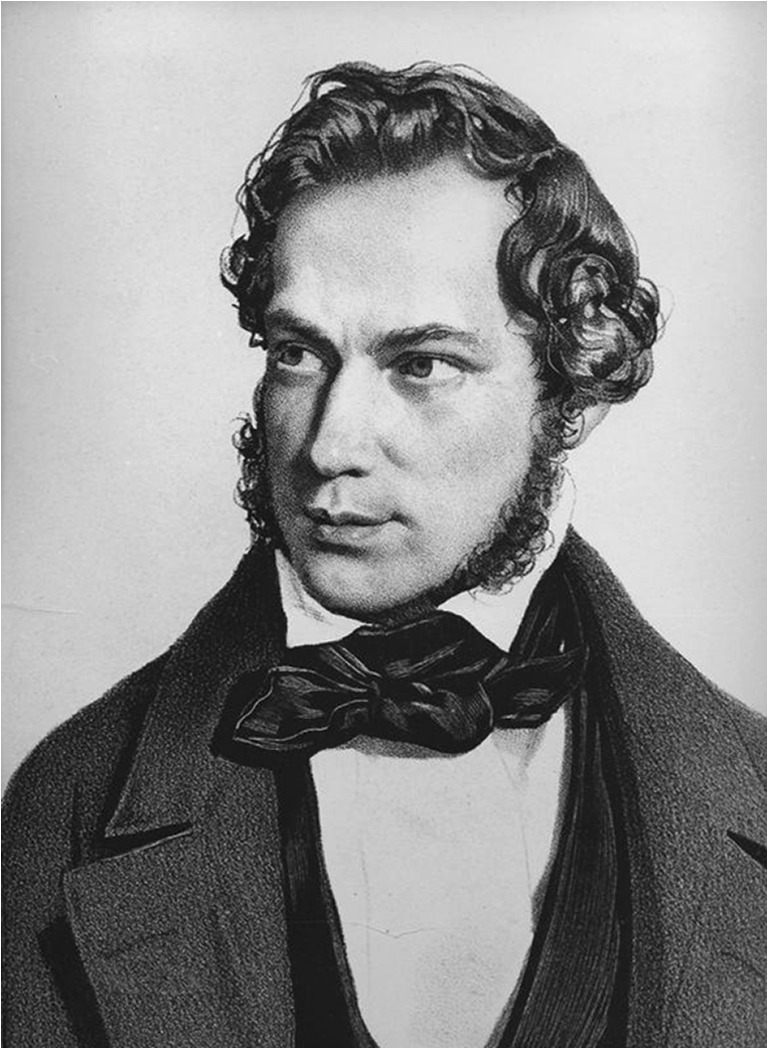
Friedrich Gustav Jakob Henle (1809–1885). Henle worked in Berlin, Zürich, Heidelberg, and Göttingen. Robert Koch was his student and together they established the Henle–Koch postulates concerning the definition of disease-causing microbes. From Images from the History of Medicine

Friedrich Henle (1809–1885) carefully dissected the microanatomy of the kidney (and many other organs) and discovered the tubular segment named after him as the loop of Henle [13]. He studied medicine at Heidelberg and Bonn, worked in anatomy in Berlin, and then became professor of anatomy in Zürich, Switzerland (Fig. 5). From there he went to Heidelberg to teach pathology, anatomy, and physiology. From Heidelberg, he published the *Handbuch der systematischen Anatomie des Menschen* [14], based on his concept that pathology and physiology were branches of one science. The cellular diversity of the tubular compartment became evident, ready to be unraveled by physiologists over the next century, starting with Carl Ludwig (1816–1895) and Claude Bernard (1813–1878), followed by Homer Smith (1895–1962), A. Newton Richards (1876–1966), Carl Gottschalk (1922–1997), and many others. Crucial for their studies were the contributions of chemists, among whom we can mention Donald van Slyke (1883–1971) and Lawrence Henderson (1878–1942), who developed techniques to allow microanalysis of plasma and urine.

Friedrich Theodor von Frerichs (professor of medicine at Berlin, 1819–1885) was perhaps the first to try to integrate clinical findings with microscopic analysis. This was taken further by his successor Friedrich von Mueller (Marburg, 1858–1941), and then by Volhard and Fahr [15].

### Renal pathology (1900–1950)

**Fig. 6:**
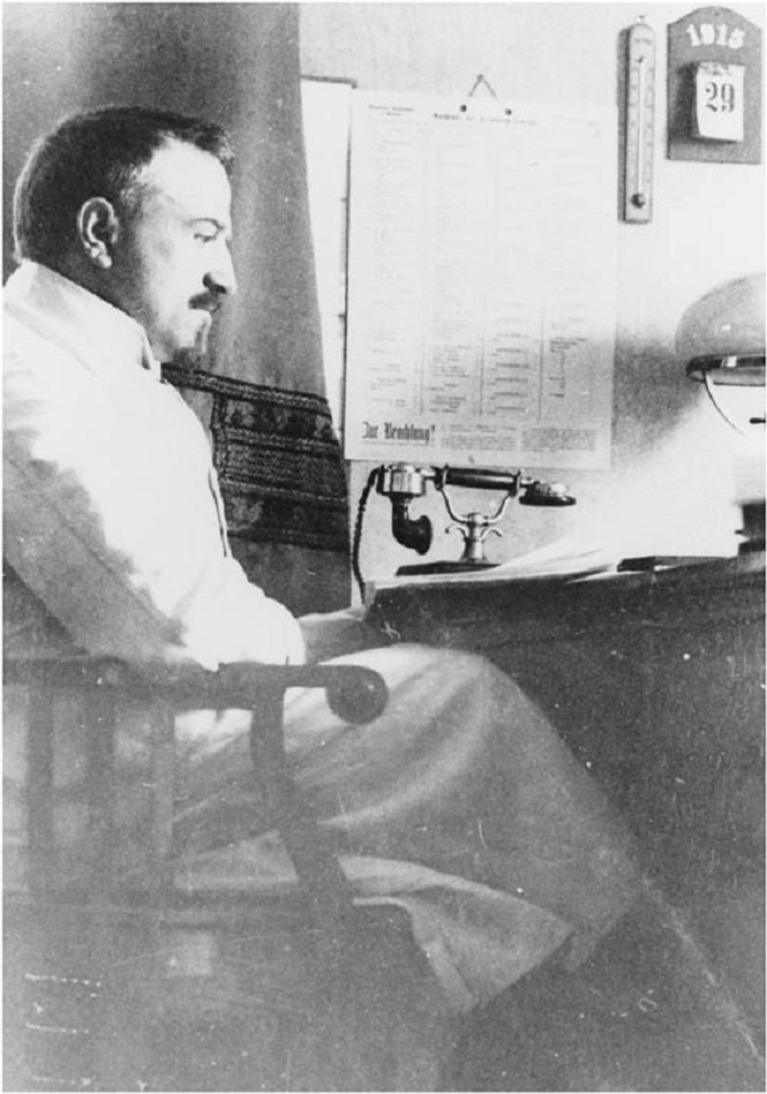
Theodor Fahr as the director of the Institute of Pathology at the University of Hamburg. From Historical Archives of the University Hospital Hamburg-Eppendorf, Institute of Medical History, University Hamburg, Germany

Theodor Fahr (1877–1945) was the first modern renal pathologist, documenting pathological changes in renal tissue with an exact eye for detail and relating these with the internist Franz Volhard to clinical expression of renal disease [15]. Fahr had trained in Giessen and received further training in Hamburg and Paris, where he worked with Ilya Metchnikoff, who in 1908 received the Nobel Prize for his work on phagocytosis. Fahr was made chairman of the institute of pathology at Hamburg University-Eppendorf in 1924 (Fig. 6).

The fruitful collaboration with Franz Volhard who worked at the university of Frankfurt-am-Main resulted in many publications, including the seminal *Die Bright’sche Nierenkrankenheit, Klinik, Pathologie und Atlas* (1914) [15] and his volume on the kidney in the series on pathological anatomy by Henke and Lubarsch [16].

In their classification of Bright’sche Nierenkrankenheiten, Fahr and Volhard classified renal disease into three major syndromes: the degenerative diseases (nephrosis), inflammatory diseases (nephritides), and chronic nephrosclerosis. Because of a lack of ultrastructural detail (the electron microscopy was not invented until 50 years later) and limited by only a superficial insight in renal inflammatory processes, the authors failed to recognize the glomerulus as the origin of proteinuria in patients with the nephrotic syndrome and placed too much emphasis on tubular reabsorption patterns coined lipoid nephrosis. However, by removing much of the confusion and controversies created by the publications of Bright and Morgagni, this new classification based on the combination of pathology and clinical expression of disease was a major improvement and became widely accepted.

Sir Arthur Ellis (1883–1966) published one of the earliest clinicopathologic studies of glomerular disease based on observations of the clinical courses of 600 patients with Bright’s disease in the London Hospital including *postmortem* histological observations in 200 patients [17]. Ellis began his career in Toronto as a clinical chemist, trained in pathology at Western Reserve University in Cleveland, and eventually became a renowned professor of medicine at the University of London and Oxford before being knighted in 1953 for his contributions to the study of kidney disease. His multidisciplinary background in clinical chemistry, pathology, and internal medicine provided a unique perspective for making clinicopathologic correlations. He correlated clinical features, associated conditions (e.g., pharyngitis), blood and urine laboratory results, and histologic findings to propose two types of nephritis. Type I nephritis had an abrupt acute onset, often following an infection, with many but not all patients recovering. Type II nephritis had a more insidious onset characterized by edema and severe proteinuria. He described and illustrated with photomicrographs distinct patterns of glomerular injury that correlated with the clinical course, including glomerular hypercellularity with numerous neutrophils in acute type I nephritis, fibrinoid necrosis, and crescents in severe type I nephritis and focal and nodular sclerosis in type II nephritis.

Further insight in the etiology and pathogenesis of renal disease was hampered by the fact that only autopsy material was available for analysis, mostly revealing no more than the endpoint of diseases, obscuring their kinetics and evolution. In addition, more knowledge was required in the field of genetics, cell biology, and immunology before renal diseases could be understood by pathologists and nephrologists as they are today. An important step forward was the introduction of experimental pathology. In a similar way as animal autopsies had preceded the precise study of the human body, laboratory animal experimentation allowed the early twentieth century scientist to induce renal disease and study its evolution over time. It permitted modulation of disease and the study of clinical symptoms. Several experimental models of renal disease were thus developed, preparing the field for a rational interpretation of renal biopsies in man.

### Experimental renal pathology

Experimental renal pathology has contributed immensely to the understanding of renal disease for over a hundred years. In 1888, Theodor Tuffier (Paris, 1857–1929) studied the effects of partial renal ablation on renal structure and function [18]. This model would be explored in detail by many investigators in the last quarter of the twentieth century, documenting the role of intracapillary hypertension, glomerular cellular injury, and accelerating loss of nephrons, as a model for progressive renal disease [19–21].

A major impetus for the understanding of renal disease was derived from studies on infection and immunity. Infectious diseases had been by far the most important threat to health and society for ages and still were around the turn of the nineteenth to twentieth century. As of the nineteenth century, clinical and experimental works successfully started to reveal the basis of infection and immunity. Around 1900, the immune response was recognized as having a dark side causing disease by inflammation, anaphylaxis, allergy, and autoimmune reactions.

These early advances in immunology received much attention, were honored with Nobel prizes, and had their immediate impact on the renal field as on every other part of medicine. The Institut Pasteur in Paris was one of the centers of research in this field. It was there that Theodor Fahr worked with Metchnikoff and where kidney disease was studied in laboratory animals exposed to anaphylactic substances [22–24].

Also at Pasteur’s Institute, W. Lindemann in 1900 developed a model of autoimmune glomerulonephritis by injecting rabbits with heterologous antiserum raised in guinea pigs against rabbit kidney [25]. This model was studied in depth by M. Masugi in 1933 [26] and was important in the new era that started in 1950. Between 1950 and 1960, *serum sickness-like glomerulonephritis* was identified as the first immune-mediated disease of the kidney caused by deposition of circulating immune complexes, a characteristic phenomenon in chronic infectious diseases and autoimmune diseases [27, 28].

## The modern era

### Renal pathology (1950–1970)

Three centuries had passed since Malpighi first observed the complex microanatomy of the renal glomerulus. Three centuries later, the almost simultaneous development of techniques to safely obtain renal tissue from a patient by means of a percutaneous needle biopsy and to analyze the tissue not only by light microscopy but also by electron microscopy and immunofluorescence microscopy suddenly provided the structural basis for diagnosis of the many different inflammatory and non-inflammatory, acute and chronic, relapsing and remitting, proteinuric and hematuric, hypertensive and non-hypertensive, and all other renal diseases that had been impossible to classify in an orderly fashion thus far. Within five decades, many questions were going to be answered, although several enigmas still remain today.

The renal needle biopsy was first used for clinical diagnosis of patients by Nils Alwall from Lund, Sweden in 1944. Alwall was successful in obtaining renal tissue by an aspiration technique from his first 13 patients but when 1 of them died from complications, he decided to no longer pursue the technique and did not publish his work until 1952 [29]. His efforts were continued by Paul Iversen and Claus Brun from Copenhagen who published their first series of renal biopsies in 1951 [30] and by Robert Kark from Chicago.

**Fig. 7:**
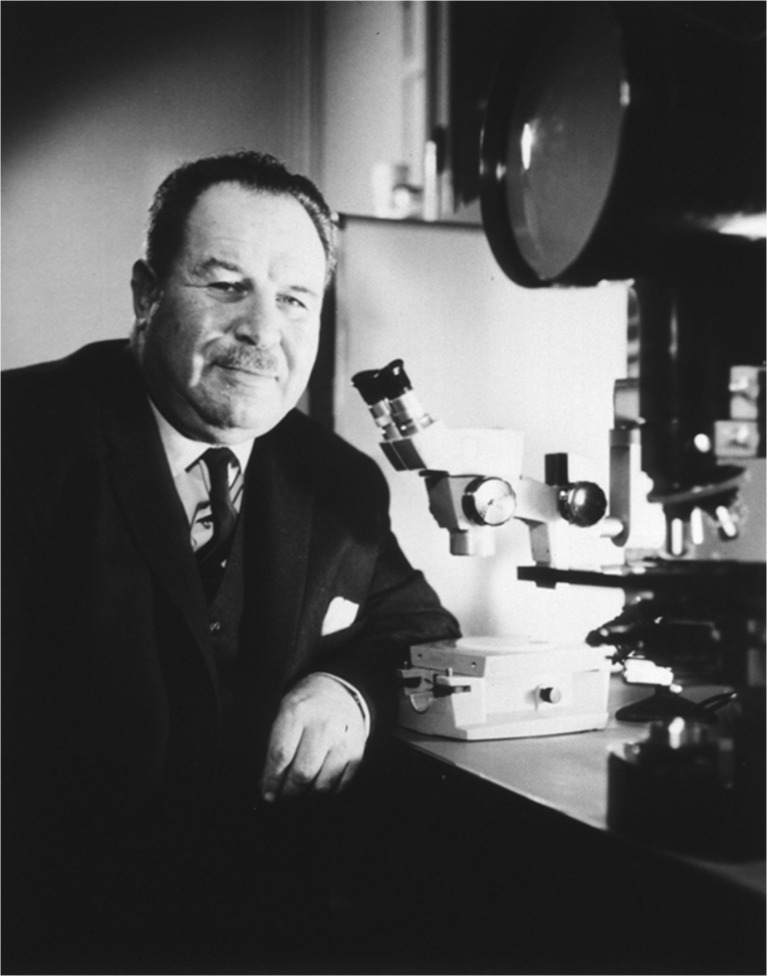
Robert M. Kark who with Robert Muehrcke and renal pathologist Conrad Pirani pioneered the use of the renal biopsy in patient care. From Images from the History of Medicine

Robert M. Kark (Fig. 7), originally from Cape Town, a renowned rugby player while a medical student at Guy’s Hospital, London, had developed an interest in nutrition during a fellowship at Harvard. In World War II, he used this expertise in nutrition to help develop K-rations and foiled a sabotage attempt on a plane to be used by Winston Churchill. Following the war, he became an expert on kidney disease at Rush-Presbyterian-St Luke's Medical Center in Chicago. Kark, who had met Iversen at a congress in Copenhagen in 1950, developed together with Muehrcke and Franklin a technique using the Vim Silverman needle (a cutting needle instead of an aspiration needle) in prone patients which proved to be much more successful than Alwall’s procedure [31]. In collaboration with one of the first modern pathologists to specialize in renal pathology, Conrad Pirani, the Chicago group exploited the new development effectively, published widely, and established the Chicago unit as a teaching center for this new technique [31, 32]. The pathology world was at first skeptical, or should we say hostile, to the idea of a tiny renal biopsy on which a full diagnosis was supposed to be made (“the smaller the biopsy, the more they want to know” is a frequently heard complaint in the practice of pathology). But several pathologists took up the challenge and successfully collaborated with nephrologists to advance the field of nephropathology.

Immunofluorescence microscopy as a technique to detect tissue-bound immune deposits had been developed by Coons and Kaplan in 1950 [33]. In 1955, Mellors first applied the technique to renal tissue [34]. Many renal diseases, particularly the various forms of glomerulonephritis, are antibody- and immune complex-mediated and the newly developed technique allowed for the first time observation of immune reactants that were contributing to the pathogenesis of many different forms of glomerulonephritis [35]. During the 1960s, Frank Dixon, Fred Germuth, and Robert McCluskey integrated the new knowledge about the pathogenesis of immune complex-mediated glomerulonephritis into concepts of diagnostic renal pathology.

Electron microscopy was new in the field as well and the first observations of ultrastructural changes in glomerular disease were published in 1957 [36].

**Fig. 8:**
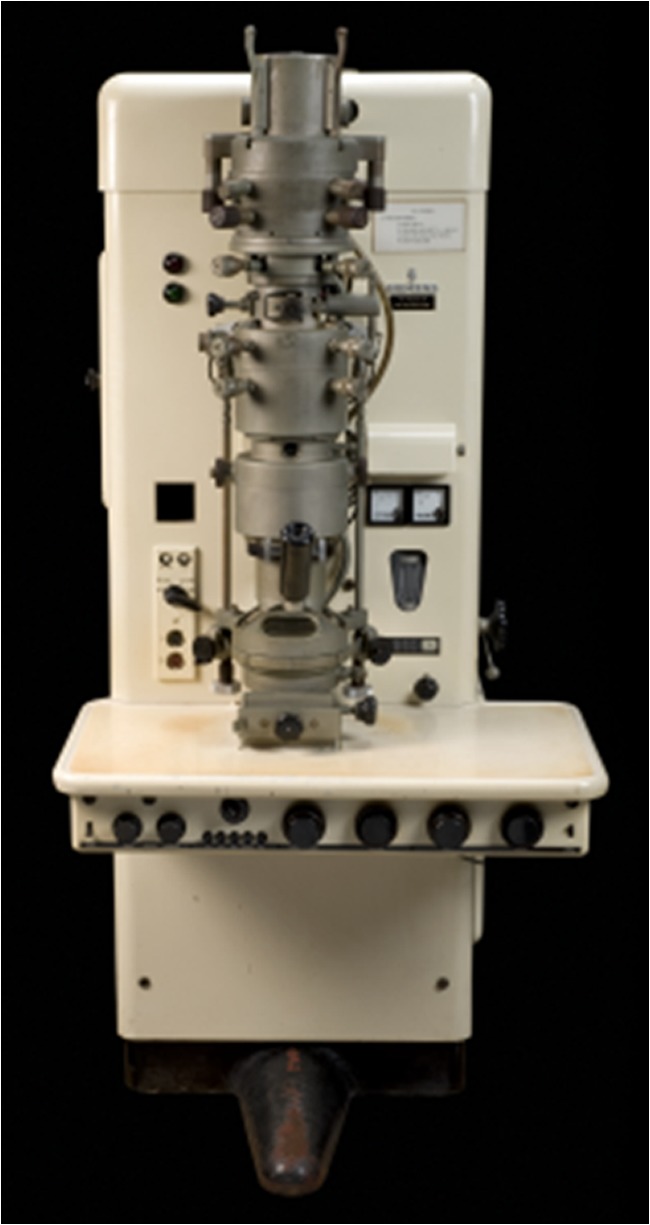
Early generation electron microscope. From Siemens, 1955; Science Museum London, UK

The fenestrated endothelium, the multiple-layered glomerular basement membrane, the mesangium, and the intricate structure of the arborizing and interdigitating podocytes were discovered by thrilled pathologists staring through the binoculars of the first generation of electron microscopes [36, 37] (Fig. 8). Conrad Pirani in Chicago, Ramzi Cotran from Boston and Jacob Churg and Edith Grishman in New York were early pioneers in applying electron microscopy to renal biopsy diagnosis.

**Fig. 9:**
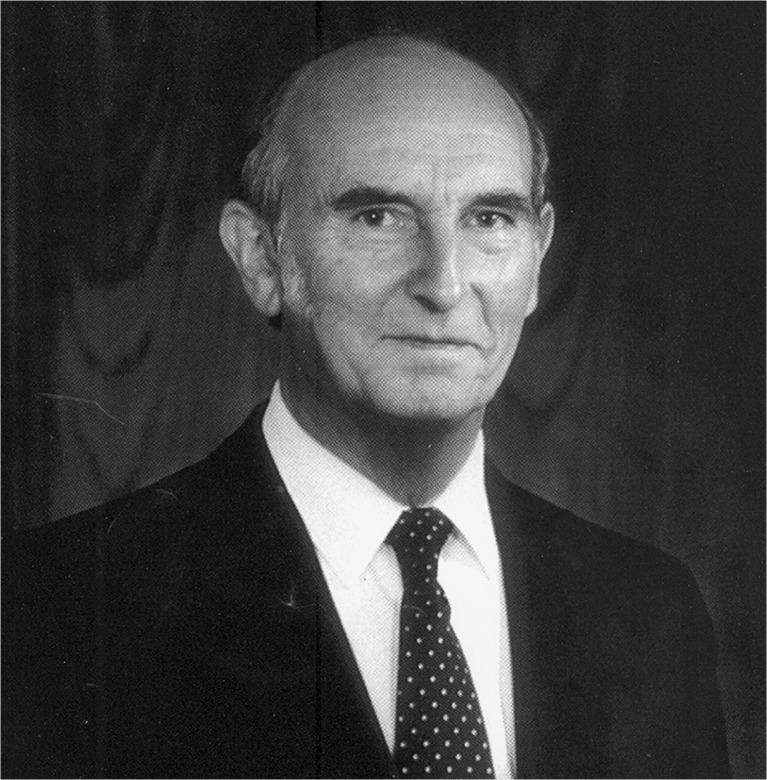
Robert Heptinstall whose ground-breaking textbook *Pathology of the Kidney* influenced generations of nephropathologists and nephrologists. From Johns Hopkins University, Baltimore MD, USA

With the advent of the renal biopsy, and the application of better histology methods and electron microscopy, the understanding of the histopathology of renal disease developed rapidly at many centers around the world. During the 1950s, the Renal Association in London had monthly meetings at the Ciba Foundation where frequent discussions of renal biopsy findings and clinical correlations were discussed. This presaged the 1961 CIBA Foundation Symposium on Renal Biopsy in London, which was a landmark event in the maturation of the field of renal pathology. The session was chaired by Arnold Rich and pioneering renal pathologists who attended included A. Bergstrand (Sweden), R. Habib (France), R.H. Heptinstall (UK), R.B. Jennings (USA), H.Z. Movat (Canada), and C.L. Pirani (USA) [38]. Knowledge of renal pathology advanced to the stage that comprehensive textbooks became available to provide guidance to nephrologists and pathologists involved in the care of patients with kidney disease. Preeminent among these was Robert H. Heptinstall’s (Fig. 9) *Pathology of the Kidney*, first published in 1966 [39], which has cataloged the progression of knowledge in nephropathology through seven editions [40]. Robert H. Heptinstall began his medical career in London as a surgeon, but after World War II he trained as a pathologist. As a young pathologist at St. Mary’s in London he collaborated with the renowned internist George Pickering on clinicopathologic studies of hypertensive nephropathy, which was the beginning of a lifelong focus on renal pathology. With radiologist John Hodson, he worked on the pathogenesis of reflux nephropathy. In 1954 he did a fellowship at Johns Hopkins with Arnold Rich and Fred Germuth studying immune complex glomerulonephritis and in 1959 with Harry Goldblatt in Cleveland. He moved permanently to USA in 1960, briefly at Washington University in St. Louis and then at Johns Hopkins for the remainder of his illustrious career. At Hopkins he continued his research on hypertensive nephropathy and also studied pyelonephritis. His lasting legacy to renal pathology is the textbook that he first published in 1966 [39]. Several valuable books on renal pathology had been published already but his was the first to emphasize observations in renal biopsies rather than autopsies. The first edition primarily covered findings by light microscopy and electron microscopy, but the second edition in 1974 included immunofluorescence microscopy observations as well as a chapter by Robert McCluskey specifically devoted to immunologic mechanisms of renal disease [41]. These books by Heptinstall heralded the current era in renal pathology.

### Renal pathology (1970 onward)

In the 1970s, the relatively few pioneers of renal pathology passed the baton to eager younger renal pathologists too numerous to name. Between 1970 and 2010, etiology, pathogenesis, clinicopathological correlations, and classification were established for renal diseases as diverse as lupus nephritis [42, 43], post-infectious glomerulonephritis [44], multiple forms of membranoproliferative glomerulonephritis [45], anti-GBM disease [46, 47], membranous glomerulopathy [48–50], IgA nephropathy [51–53], ANCA-associated vasculitis and glomerulonephritis [54–56], focal segmental glomerulosclerosis and other podocytopathies [57–59], hereditary renal diseases [47], hemolytic uremic syndrome [60], tubulointerstitial disease [61], renal injury in the kidney transplant patient [62], and many others (Table 1). Today’s renal pathologists continue to build on the strong foundation laid by the pioneers of renal pathology to advance our knowledge of the pathology and pathogenesis of kidney diseases and to use this to improve the management of patients.

### Perspective

In the almost 100 years since the pioneering clinicopathologic studies of Volhard the internist and Fahr the pathologist, tremendous advances have been made in our understanding of the pathology and pathophysiology of kidney disease and the care of patients with kidney disease. These advances have come as a result of intense collaboration between nephrologists and nephropathologists and have been facilitated by sharing new knowledge disseminated through national and international scientific societies, journals, and annual congresses in the field of pathology and nephrology. Advances in renal pathology also have been fostered in particular by the vitality of the Renal Pathology Society, which is international, and the Nephropathology Working Group of the European Society of Pathology. International collaborative efforts to benefit from advances in nephropathology have not been confined to economically well-off countries but also have been extended to the developing countries through the International Society of Nephrology [63]. Reviewing the history of renal pathology reveals how an entire field can only advance by the contributions of many, through implementing new technical developments, and fostered by the fortune of economic prosperity.
